# Deep vein thrombosis and pulmonary embolus associated with a ruptured popliteal aneurysm – a cautionary note

**DOI:** 10.1186/1749-7922-2-34

**Published:** 2007-12-20

**Authors:** Pandanaboyana Sanjay, Mike H Lewis

**Affiliations:** 1 Department of Surgery, Royal Glamorgan Hospital, Llantrisant, Wales, UK

## Abstract

Popliteal artery aneurysms representing 80% of peripheral artery aneurysms rarely rupture (a reported incidence of 0.1–2.8 %) and second commonest in frequency after aorto-iliac aneurysms. They usually present with pain, swelling, occlusion or distal embolisation and can cause diagnostic difficulties. We report a 78 year old man who was previously admitted to hospital with a pulmonary embolus secondary to deep venous thrombosis. He was heparinized then warfarinised and was readmitted with a ruptured popliteal aneurysm leading to a large pseudo aneurysm formation. The pulmonary embolus had been due to popliteal vein thrombosis and propagation of the clot. A thorough review of literature identified only one previously reported case of ruptured popliteal artery aneurysm and subsequent large pseudo aneurysm formation. We feel it is important to exclude a popliteal aneurysm in a patient with DVT. This may be more common than the published literature suggests.

## Introduction

Popliteal artery aneurysms even in busy vascular units are infrequent and difficult to manage. Ruptured popliteal aneurysms are extremely rare and are said to have varied presentations and can cause diagnostic dilemma. We present a 78 year old man who presented with a ruptured popliteal aneurysm associated with deep vein thrombosis and pulmonary embolism. A through literature search identified only one previously reported case of ruptured popliteal artery aneurysm with large psuedoaneurysm formation with deep vein thrombosis [1].

## Case presentation

A 78 year old Caucasian male presented to our casualty 6 weeks following admission elsewhere with a history of swelling of the right leg and a pulmonary embolus. He had undergone VQ scanning with a proven diagnosis of pulmonary embolus and was therefore anticoagulated with heparin and warfarin.

On admission he had swelling of the right knee and lower thigh with loss of sensation on the dorsolateral aspect of the right foot. Clinical examination revealed a large mass in the right popliteal fossa. The initial diagnosis was that of deep venous thrombosis and imaging was undertaken and the patient referred for a vascular opinion.

**Figure 1:**
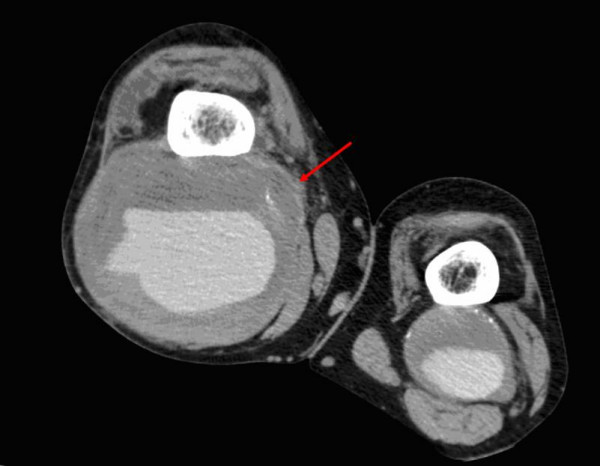
CT image showing bilateral popliteal artery aneurysms with extravasation of contrast-enhanced blood with surrounding aneurysm on the right side. (Arrow pointing to the popliteal artery).

**Figure 2:**
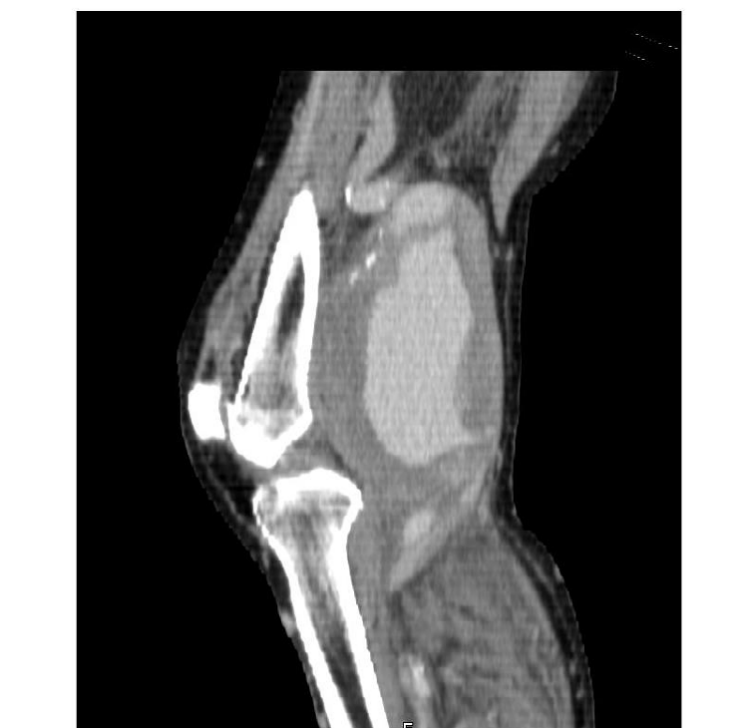
CT lateral projection showing the popliteal artery aneurysm with the pseudoaneurysm.

On vascular review, the patient had a pulsatile mass comparable with a popliteal artery aneurysm of 12 cms diameter on the right and 6 cms on the left. His foot was warm and well perfused and his INR was 2.6. He underwent a duplex scan of the lower limb arteries which revealed ectatic iliacs and an abdominal aorta of 3.4 cms in diameter. A further CT arteriogram (fig 1, 2) confirmed that in fact the swelling on the right side was indeed a pseudo aneurysm following rupture with peripheral calcification.

The dilemma was therefore a patient with an established pulmonary embolus and a friable clot in the iliac veins, anticoagulation and the treatment of the ruptured aneurysm. Due to lack of expertise we elected to continue with full anticoagulation rather than place an inferior vena caval filter. Operative exploration was performed through a medial approach under full anticoagulation and the right popliteal artery was found to contain a large volume of blood pointing laterally. The popliteal aneurysm was excluded. Duplex examination of the upper and lower limb veins revealed that the veins were of small diameter therefore revascularization of the leg was established by an inlay 8 mm PTFE graft. Due to continuous oozing, the aneurysm sac was packed and the end of the pack brought out laterally. The pack was removed at 48 hours under sedation. The patient made an uneventful recovery. Six weeks later he underwent repair of the left popliteal aneurysm.

## Discussion

Popliteal artery aneurysms account for 2/3rds of all peripheral artery aneurysms. They mostly occur in the seventh decade of life and have a very high male preponderance of around 30:1 [2]. The aetiology of popliteal artery aneurysms is varied with atherosclerosis being the commonest cause. The other causative factors include trauma, radiological interventions including PTA and post stenotic aneurysms secondary to entrapment. Mycotic and syphilitic aneurysms have become very rare.

Popliteal aneurysms are generally associated with poor prognosis. Death and limb loss have both been reported, and approximately 1% of the patients will be left with residual symptoms [3] Though popliteal artery is the most common site of aneurysm formation after the aortoiliac system, the reported incidences of popliteal artery rupture is very low (0.1–2.8 %) [4]. The possible reasons for such a low rupture rate could due to the fact that the popliteal artery is surrounded by musculofascial and bony structures offering resistance and protection.

The clinical presentation of ruptured popliteal aneurysms varies widely. The characteristic presentation is said to be a swelling behind the knee associated with acute pain. They can also present as painless progressive enlargement of the leg, swollen leg with anaemia or with deep vein thrombosis (DVT) [5]. Deep venous thrombosis is usually due to venous compression, and very rarely can result in thromboembolic phenomenon as was evident in our case. In our case the right popliteal artery aneurysm ruptured resulting in a haematoma, which was limited by the surrounding tissues. It was starting to point laterally but cultures of the blood were negative. Local venous stasis and compressive effects resulted in deep venous thrombosis of the popliteal veins, which resulted in the pulmonary embolus.

Ultrasound examination is of limited value in a case of suspected rupture; however it does help in confirming the presence of an aneurysm. CT scan with or without angiography is the investigation of choice to confirm popliteal artery rupture [6]. It can clearly demonstrate the presence of the aneurysm and the associated haematoma making the diagnosis obvious. Angiography is helpful to identify distal runoff and the site for distal anastomosis.

It is stated that ruptured popliteal artery aneurysms are best treated by ligation and continuity established preferably by autologous saphenous vein graft or by synthetic grafts when the patients have unsuitable veins. More recently this condition has been managed by percutaneous endovascular treatment in medically unfit patients [7].

Since there is no evidence to show that routine screening of popliteal arteries will detect asymptomatic aneurysms [8] and in addition the pick up rate is low, the majority of the patients with popliteal artery aneurysms present with limb threatening complications including thrombosis and distal embolisation. Venous occlusion has been documented [9] but our case shows that DVT and PE is another poorly documented presentation. A high index of suspicion is needed in a patient presenting with signs and symptoms of deep vein thrombosis or a massively swollen leg with impending compartment syndrome or anaemia with out any clear diagnosis. Inappropriate management of patients with deep venous thrombosis from unrecognised arterial aneurysms is associated with unacceptable morbidity and mortality.

## Key points

**1. **Popliteal artery aneurysms very rarely rupture (a reported incidence of 0.1–2.8 %).

**2. **Ruptured popliteal aneurysms have varied presentations and can cause diagnostic dilemma.

**3. **A high index of suspicion is needed in a patient presenting with signs and symptoms of deep vein thrombosis or a massively swollen leg with impending compartment syndrome or anaemia with out any clear diagnosis

**4. **Inappropriate management of patients with deep venous thrombosis from unrecognised arterial aneurysms is associated with unacceptable morbidity and mortality.
